# Supplementary material for: Widespread Epistasis between Cancer Driver Mutations and Allele-Specific Copy Number Variations
Source: bioRxiv. 2025 Dec 14:2025.12.11.693698. Preprint. [Version 1] doi: 10.64898/2025.12.11.693698 (PMC12710650; doi:10.64898/2025.12.11.693698)
Supplement: Supplement 1 — Supplementary Figure 1: Observed Proportions, Homozygous Deletions, and eccDNA Impacts of Co-occurring Mutations and CNVs (A) Significant gene-cancer pairs from the TCGA datasets, showing distinct mutations across all samples and observed proportion for mutation and CNV co-occurrence within the cancer cohort, where only the gene-cancer pairs with the highest proportions are shown. (B) For genes co-occurring with CND, genes with the most homozygous deletions are shown, matched by the percentage of mutations in the same gene by cancer. (C) Patients with mutations and copy number alterations (MUT+CNA) who also have eccDNA fragments containing the given gene. (D) Samples with mutations impacted by CNA (MUT+ CNA), samples with eccDNA fragments containing a full gene, and the intersection where samples have both. Supplementary Figure 2: Correlating RNA Allelic Imbalance and Total Gene Expression (A)-(D) – Correlation for significant genes co-occurring with CNA, where each data point reflects a sample with co-occurring mutations and CNA. (E)-(H) - Correlation for significant genes co-occurring with CND, where each data point reflects a sample with co-occurring mutations and CND. Supplementary Figure 3: DNA Allelic Imbalance of the Most Frequently Observed Mutations that Co-Occurred with CNVs A-O: Single genes with the commonest mutations associated with CNA/CND across all cancer types. Each figure shows DNA read counts for all the commonest mutations with each point being a single mutation. The highest and lowest VAF mutations are labelled. Mutations are colored by cancer and density plots show VAF for mutations without CNVs. Supplementary Figure 4: RNA Allelic Imbalance of the Most Frequently Observed Mutations that Co-Occurred with CNVs A-N: Single genes with the commonest mutations associated with CNA/CND across all cancer types. Each figure shows RNA read counts for all the commonest mutations with each point being a single mutation. The highest and lowest VAF mutation [file media-1.pdf]

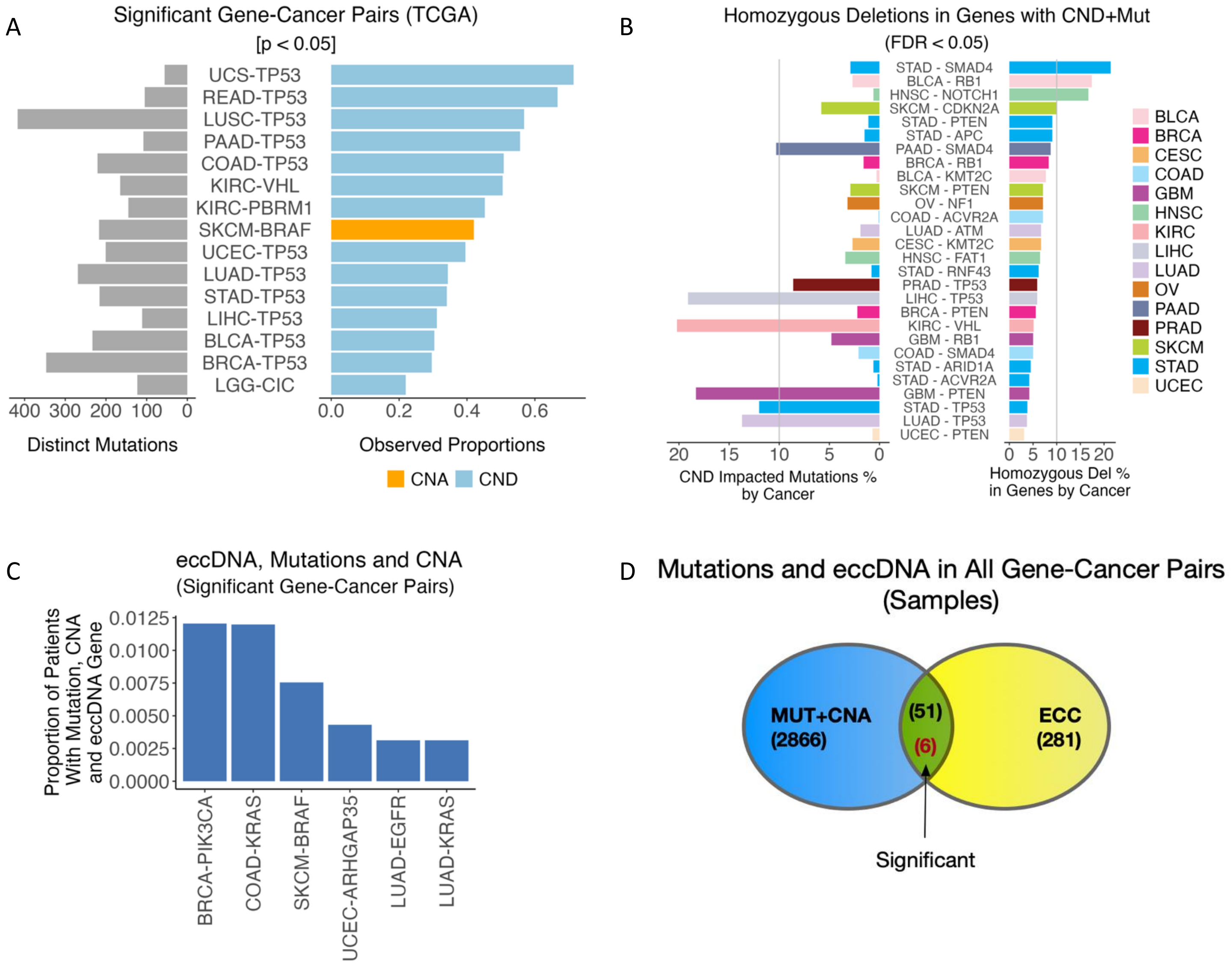
A
Significant Gene-Cancer Pairs (TCGA)
[p < 0.05]
UCS-TP53
READ-TP53
LUSC-TP53
PAAD-TP53
COAD-TP53
KIRC-VHL
KIRC-PBRM1
SKCM-BRAF
UCEC-TP53
LUAD-TP53
STAD-TP53
LIHC-TP53
BLCA-TP53
BRCA-TP53
LGG-CIC
400 300 200 100 0
Distinct Mutations
0.0 0.2 0.4 0.6
Observed Proportions
CNA CND
B
Homozygous Deletions in Genes with CND+Mut
(FDR < 0.05)
STAD - SMAD4
BLCA - RB1
HNSC - NOTCH1
SKCM - CDKN2A
STAD - PTEN
STAD - APC
PAAD - SMAD4
BRCA - RB1
BLCA - KMT2C
SKCM - PTEN
OV - NF1
COAD - ACVR2A
LUAD - ATM
CESC - KMT2C
HNSC - FAT1
STAD - RNF43
PRAD - TP53
LIHC - TP53
BRCA - PTEN
KIRC - VHL
GBM - RB1
COAD - SMAD4
STAD - ARID1A
STAD - ACVR2A
GBM - PTEN
STAD - TP53
LUAD - TP53
UCEC - PTEN
20 15 10 5 0
CND Impacted Mutations % by Cancer
0 5 10 15 20
Homozygous Del % in Genes by Cancer
BLCA
BRCA
CESC
COAD
GBM
HNSC
KIRC
LIHC
LUAD
OV
PAAD
PRAD
SKCM
STAD
UCEC
C
eccDNA, Mutations and CNA
(Significant Gene-Cancer Pairs)
Proportion of Patients With Mutation, CNA and eccDNA Gene
0.0125
0.0100
0.0075
0.0050
0.0025
0.0000
BRCA-PIK3CA
COAD-KRAS
SKCM-BRAF
UCEC-ARHGAP35
LUAD-EGFR
LUAD-KRAS
D
Mutations and eccDNA in All Gene-Cancer Pairs
(Samples)
MUT+CNA
(2866)
(51)
(6)
ECC
(281)
Significant

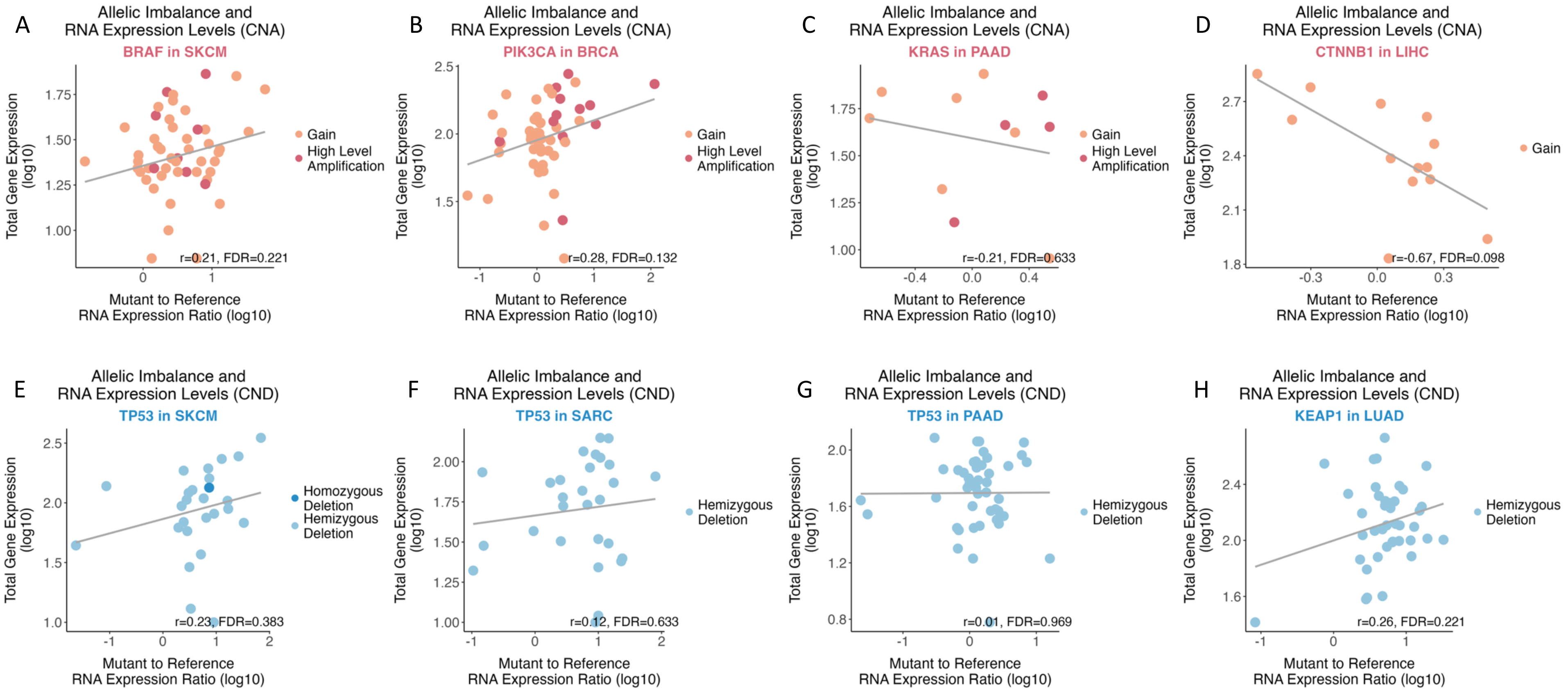
A
Allelic Imbalance and
RNA Expression Levels (CNA)
BRAF in SKCM
Total Gene Expression (log10)
Mutant to Reference RNA Expression Ratio (log10)
Gain
High Level Amplification
r=0.21, FDR=0.221
B
Allelic Imbalance and
RNA Expression Levels (CNA)
PIK3CA in BRCA
Total Gene Expression (log10)
Mutant to Reference RNA Expression Ratio (log10)
Gain
High Level Amplification
r=0.28, FDR=0.132
C
Allelic Imbalance and
RNA Expression Levels (CNA)
KRAS in PAAD
Total Gene Expression (log10)
Mutant to Reference RNA Expression Ratio (log10)
Gain
High Level Amplification
r=-0.21, FDR=0.633
D
Allelic Imbalance and
RNA Expression Levels (CNA)
CTNNB1 in LIHC
Total Gene Expression (log10)
Mutant to Reference RNA Expression Ratio (log10)
Gain
r=-0.67, FDR=0.098
E
Allelic Imbalance and
RNA Expression Levels (CND)
TP53 in SKCM
Total Gene Expression (log10)
Mutant to Reference RNA Expression Ratio (log10)
Homozygous Deletion
Hemizygous Deletion
r=0.23, FDR=0.383
F
Allelic Imbalance and
RNA Expression Levels (CND)
TP53 in SARC
Total Gene Expression (log10)
Mutant to Reference RNA Expression Ratio (log10)
Hemizygous Deletion
r=0.12, FDR=0.633
G
Allelic Imbalance and
RNA Expression Levels (CND)
TP53 in PAAD
Total Gene Expression (log10)
Mutant to Reference RNA Expression Ratio (log10)
Hemizygous Deletion
r=0.01, FDR=0.969
H
Allelic Imbalance and
RNA Expression Levels (CND)
KEAP1 in LUAD
Total Gene Expression (log10)
Mutant to Reference RNA Expression Ratio (log10)
Hemizygous Deletion
r=0.26, FDR=0.221

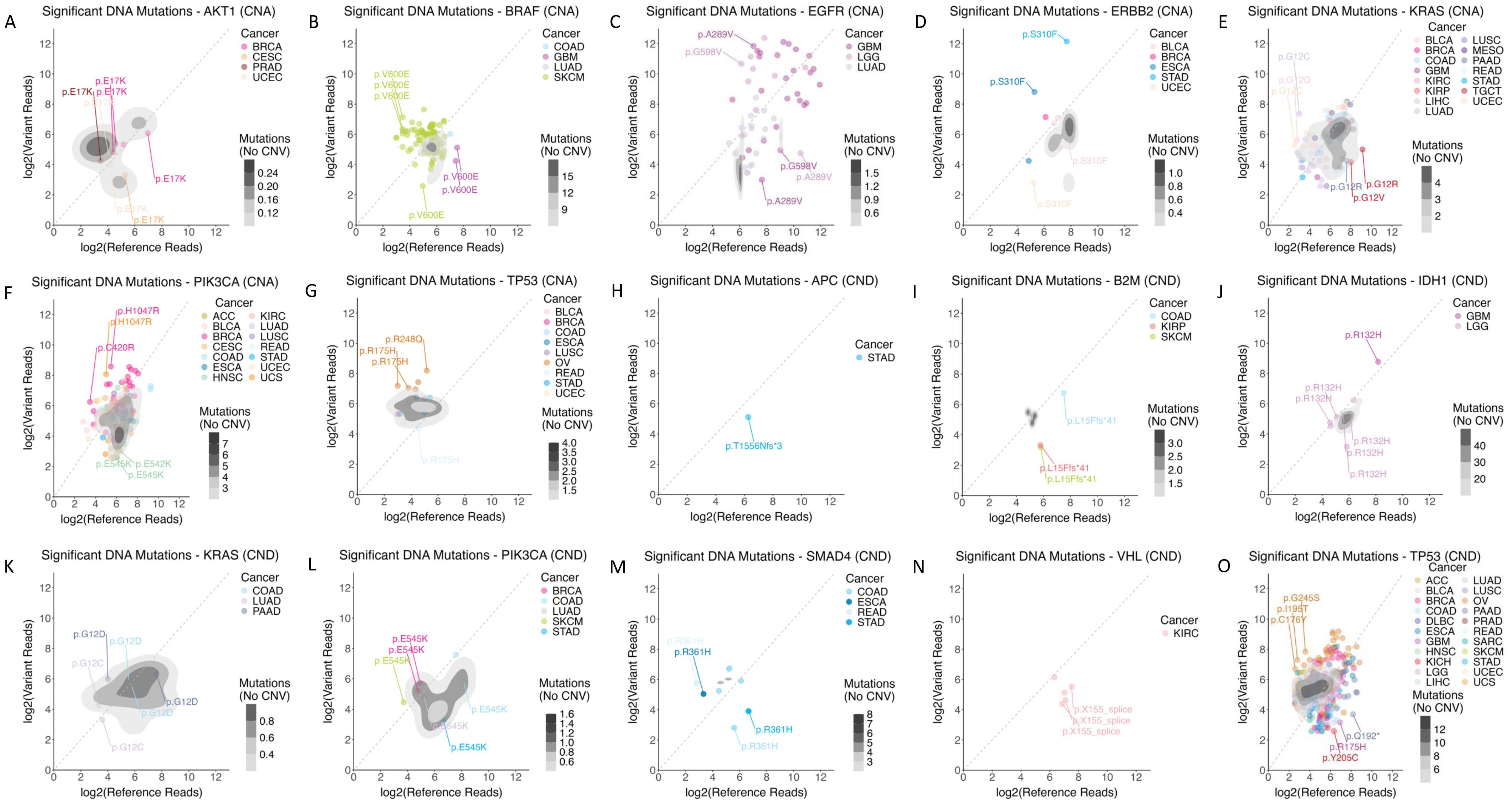

A
Significant DNA Mutations - AKT1 (CNA)
B
Significant DNA Mutations - BRAF (CNA)
C
Significant DNA Mutations - EGFR (CNA)
D
Significant DNA Mutations - ERBB2 (CNA)
E
Significant DNA Mutations - KRAS (CNA)
F
Significant DNA Mutations - PIK3CA (CNA)
G
Significant DNA Mutations - TP53 (CNA)
H
Significant DNA Mutations - APC (CND)
I
Significant DNA Mutations - B2M (CND)
J
Significant DNA Mutations - IDH1 (CND)
K
Significant DNA Mutations - KRAS (CND)
L
Significant DNA Mutations - PIK3CA (CND)
M
Significant DNA Mutations - SMAD4 (CND)
N
Significant DNA Mutations - VHL (CND)
O
Significant DNA Mutations - TP53 (CND)
log2(Variant Reads)
log2(Reference Reads)
Cancer
Mutations (No CNV)

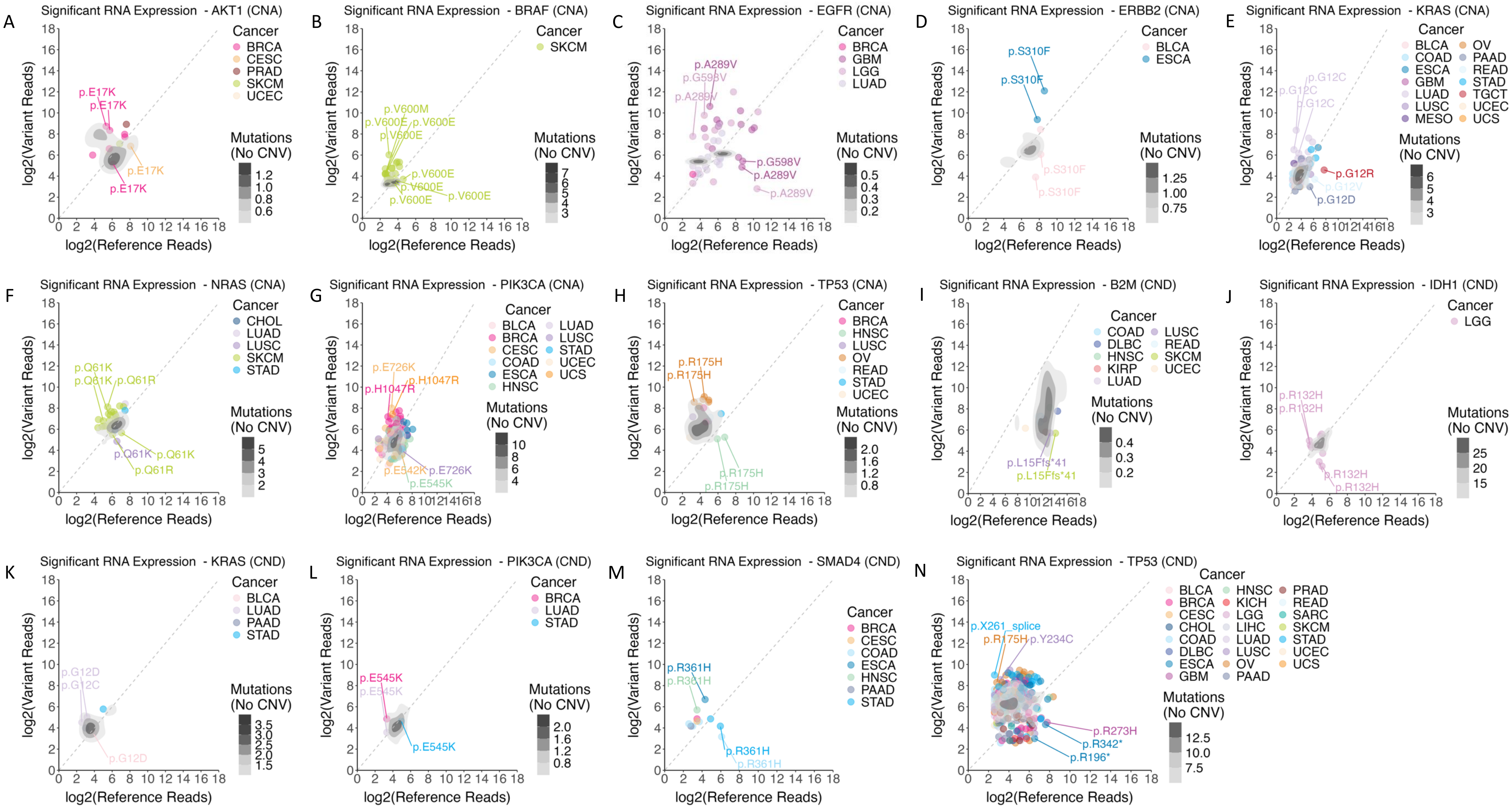
A
Significant RNA Expression - AKT1 (CNA)
Cancer
BRCA
CESC
PRAD
SKCM
UCEC
Mutations
(No CNV)
1.2
1.0
0.8
0.6
p.E17K
p.E17K
p.E17K
p.E17K
log2(Variant Reads)
log2(Reference Reads)
B
Significant RNA Expression - BRAF (CNA)
Cancer
SKCM
Mutations
(No CNV)
7
6
5
4
3
p.V600M
p.V600E
p.V600E
p.V600E
p.V600E
p.V600E
p.V600E
p.V600E
log2(Variant Reads)
log2(Reference Reads)
C
Significant RNA Expression - EGFR (CNA)
Cancer
BRCA
GBM
LGG
LUAD
Mutations
(No CNV)
0.5
0.4
0.3
0.2
p.A289V
p.G598V
p.A289V
p.G598V
p.A289V
p.A289V
log2(Variant Reads)
log2(Reference Reads)
D
Significant RNA Expression - ERBB2 (CNA)
Cancer
BLCA
ESCA
Mutations
(No CNV)
1.25
1.00
0.75
p.S310F
p.S310F
p.S310F
p.S310F
log2(Variant Reads)
log2(Reference Reads)
E
Significant RNA Expression - KRAS (CNA)
Cancer
BLCA
COAD
ESCA
GBM
LUAD
LUSC
MESO
OV
PAAD
READ
STAD
TGCT
UCEC
UCS
Mutations
(No CNV)
6
5
4
3
p.G12C
p.G12C
p.G12C
p.G12R
p.G12V
p.G12D
log2(Variant Reads)
log2(Reference Reads)
F
Significant RNA Expression - NRAS (CNA)
Cancer
CHOL
LUAD
LUSC
SKCM
STAD
Mutations
(No CNV)
5
4
3
2
p.Q61K
p.Q61K
p.Q61R
p.Q61K
p.Q61K
p.Q61R
log2(Variant Reads)
log2(Reference Reads)
G
Significant RNA Expression - PIK3CA (CNA)
Cancer
BLCA
BRCA
CESC
COAD
ESCA
HNSC
LUAD
LUSC
STAD
UCEC
UCS
Mutations
(No CNV)
10
8
6
4
p.E726K
p.H1047R
p.H1047R
p.E542K
p.E726K
p.E545K
log2(Variant Reads)
log2(Reference Reads)
H
Significant RNA Expression - TP53 (CNA)
Cancer
BRCA
HNSC
LUSC
OV
READ
STAD
UCEC
Mutations
(No CNV)
2.0
1.6
1.2
0.8
p.R175H
p.R175H
p.R175H
p.R175H
log2(Variant Reads)
log2(Reference Reads)
I
Significant RNA Expression - B2M (CND)
Cancer
COAD
DLBC
HNSC
KIRP
LUAD
LUSC
READ
SKCM
UCEC
Mutations
(No CNV)
0.4
0.3
0.2
p.L15Ffs*41
p.L15Ffs*41
log2(Variant Reads)
log2(Reference Reads)
J
Significant RNA Expression - IDH1 (CND)
Cancer
LGG
Mutations
(No CNV)
25
20
15
p.R132H
p.R132H
p.R132H
p.R132H
log2(Variant Reads)
log2(Reference Reads)
K
Significant RNA Expression - KRAS (CND)
Cancer
BLCA
LUAD
PAAD
STAD
Mutations
(No CNV)
3.5
3.0
2.5
2.0
1.5
p.G12D
p.G12C
p.G12D
log2(Variant Reads)
log2(Reference Reads)
L
Significant RNA Expression - PIK3CA (CND)
Cancer
BRCA
LUAD
STAD
Mutations
(No CNV)
2.0
1.6
1.2
0.8
p.E545K
p.E545K
p.E545K
log2(Variant Reads)
log2(Reference Reads)
M
Significant RNA Expression - SMAD4 (CND)
Cancer
BRCA
CESC
COAD
ESCA
HNSC
PAAD
STAD
p.R361H
p.R361H
p.R361H
p.R361H
log2(Variant Reads)
log2(Reference Reads)
N
Significant RNA Expression - TP53 (CND)
Cancer
BLCA
BRCA
CESC
CHOL
COAD
DLBC
ESCA
GBM
HNSC
KICH
LGG
LIHC
LUAD
LUSC
OV
PAAD
PRAD
READ
SARC
SKCM
STAD
UCEC
UCS
Mutations
(No CNV)
12.5
10.0
7.5
p.X261_splice
p.R175H
p.Y234C
p.R273H
p.R342*
p.R196*
log2(Variant Reads)
log2(Reference Reads)

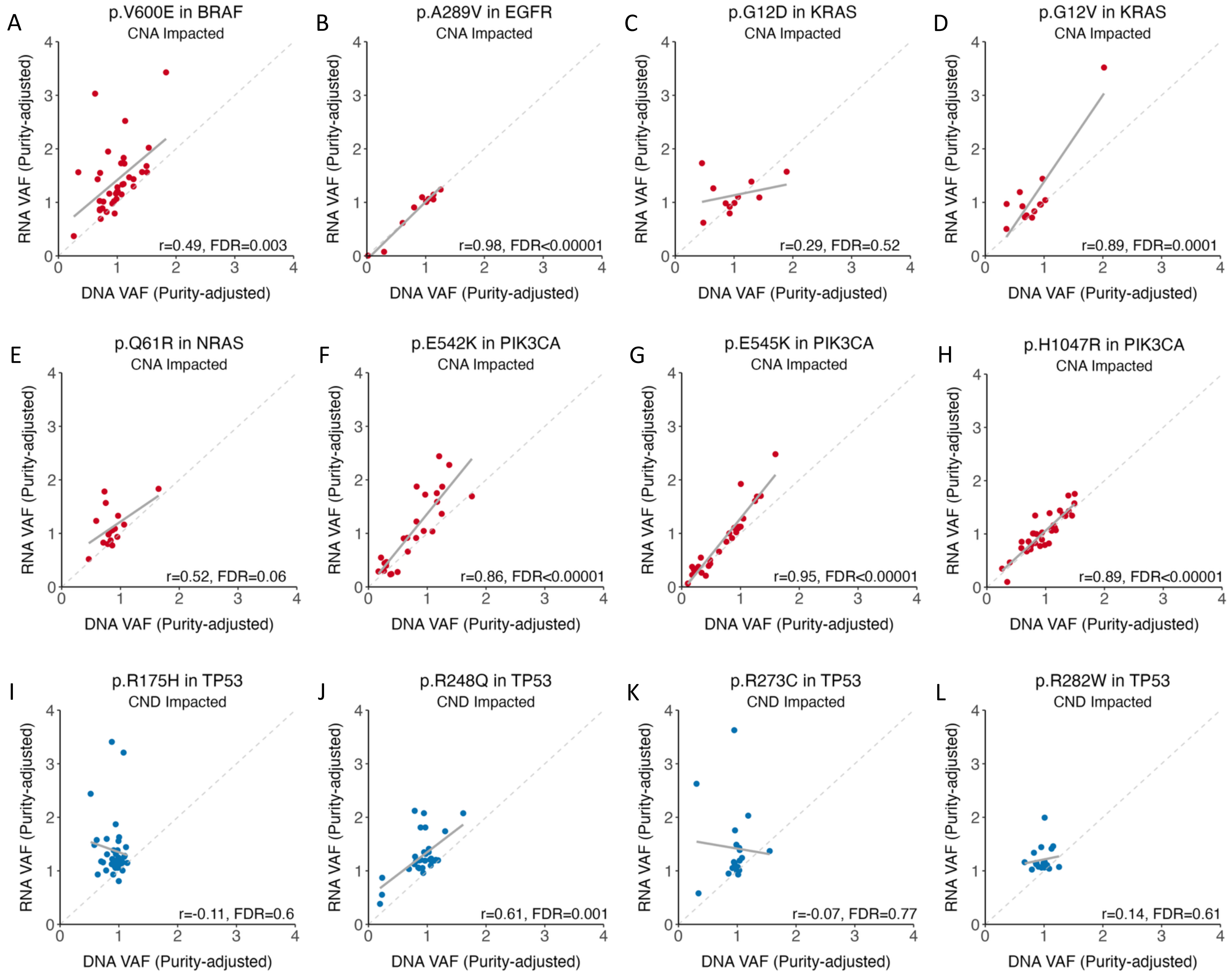

A
p.V600E in BRAF
CNA Impacted
r=0.49, FDR=0.003
B
p.A289V in EGFR
CNA Impacted
r=0.98, FDR<0.00001
C
p.G12D in KRAS
CNA Impacted
r=0.29, FDR=0.52
D
p.G12V in KRAS
CNA Impacted
r=0.89, FDR=0.0001
E
p.Q61R in NRAS
CNA Impacted
r=0.52, FDR=0.06
F
p.E542K in PIK3CA
CNA Impacted
r=0.86, FDR<0.00001
G
p.E545K in PIK3CA
CNA Impacted
r=0.95, FDR<0.00001
H
p.H1047R in PIK3CA
CNA Impacted
r=0.89, FDR<0.00001
I
p.R175H in TP53
CND Impacted
r=-0.11, FDR=0.6
J
p.R248Q in TP53
CND Impacted
r=0.61, FDR=0.001
K
p.R273C in TP53
CND Impacted
r=-0.07, FDR=0.77
L
p.R282W in TP53
CND Impacted
r=0.14, FDR=0.61
RNA VAF (Purity-adjusted)
DNA VAF (Purity-adjusted)

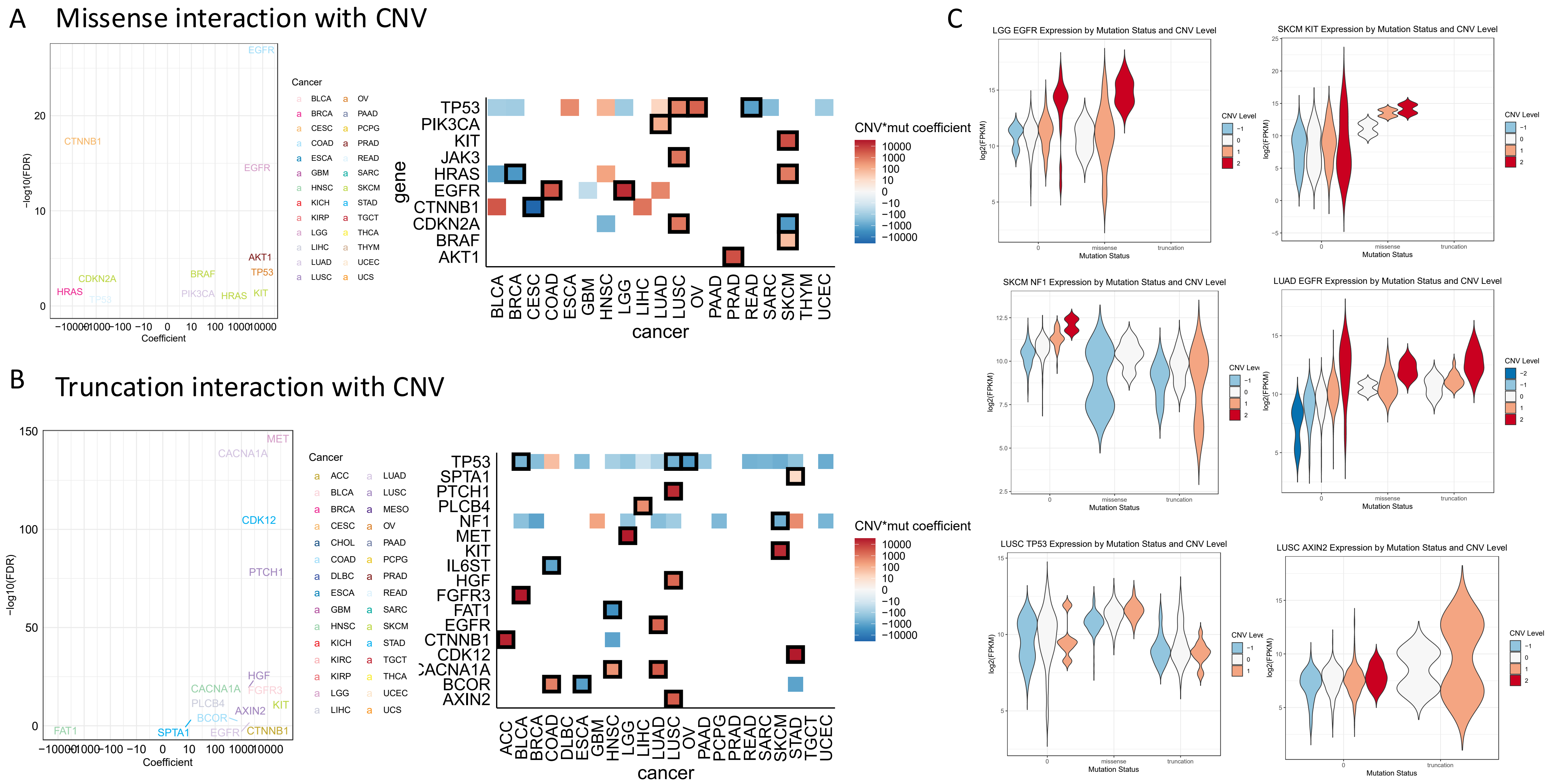
A
Missense interaction with CNV
B
Truncation interaction with CNV
C
−log10(FDR)
Coefficient
Cancer
BLCA
BRCA
CESC
COAD
ESCA
GBM
HNSC
KICH
KIRP
LGG
LIHC
LUAD
LUSC
OV
PAAD
PCPG
PRAD
READ
SARC
SKCM
STAD
TGCT
THCA
THYM
UCEC
UCS
ACC
CHOL
DLBC
KIRC
MESO
gene
cancer
CNV*mut coefficient
10000
1000
100
10
0
−10
−100
−1000
−10000
TP53
PIK3CA
KIT
JAK3
HRAS
EGFR
CTNNB1
CDKN2A
BRAF
AKT1
SPTA1
PTCH1
PLCB4
NF1
MET
IL6ST
HGF
FGFR3
FAT1
CDK12
CACNA1A
BCOR
AXIN2
LGG EGFR Expression by Mutation Status and CNV Level
SKCM KIT Expression by Mutation Status and CNV Level
SKCM NF1 Expression by Mutation Status and CNV Level
LUAD EGFR Expression by Mutation Status and CNV Level
LUSC TP53 Expression by Mutation Status and CNV Level
LUSC AXIN2 Expression by Mutation Status and CNV Level
log2(FPKM)
Mutation Status
missense
truncation
CNV Level

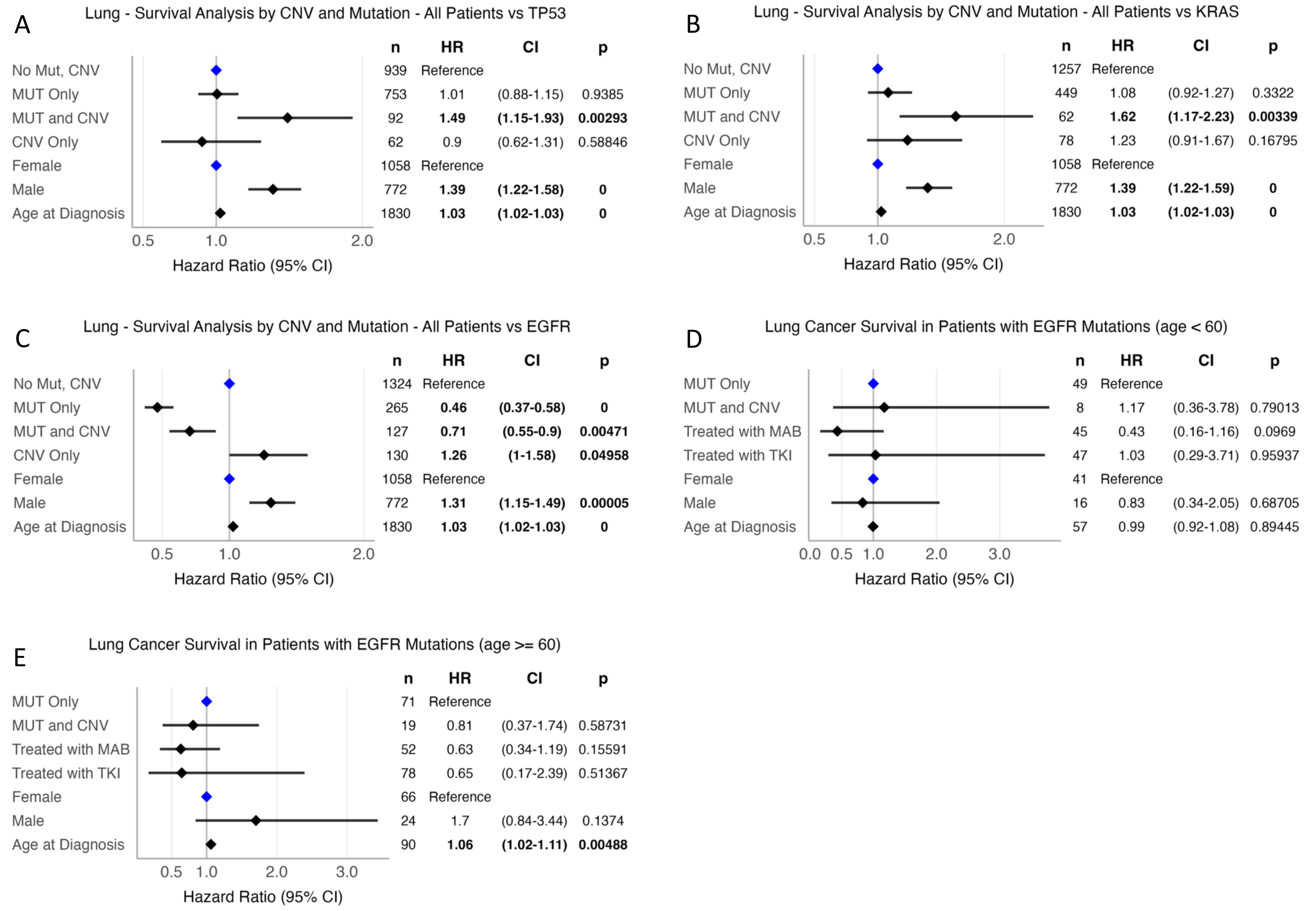
A
Lung - Survival Analysis by CNV and Mutation - All Patients vs TP53
n HR CI p
No Mut, CNV 939 Reference
MUT Only 753 1.01 (0.88-1.15) 0.9385
MUT and CNV 92 1.49 (1.15-1.93) 0.00293
CNV Only 62 0.9 (0.62-1.31) 0.58846
Female 1058 Reference
Male 772 1.39 (1.22-1.58) 0
Age at Diagnosis 1830 1.03 (1.02-1.03) 0
0.5 1.0 2.0
Hazard Ratio (95% CI)
B
Lung - Survival Analysis by CNV and Mutation - All Patients vs KRAS
n HR CI p
No Mut, CNV 1257 Reference
MUT Only 449 1.08 (0.92-1.27) 0.3322
MUT and CNV 62 1.62 (1.17-2.23) 0.00339
CNV Only 78 1.23 (0.91-1.67) 0.16795
Female 1058 Reference
Male 772 1.39 (1.22-1.59) 0
Age at Diagnosis 1830 1.03 (1.02-1.03) 0
0.5 1.0 2.0
Hazard Ratio (95% CI)
C
Lung - Survival Analysis by CNV and Mutation - All Patients vs EGFR
n HR CI p
No Mut, CNV 1324 Reference
MUT Only 265 0.46 (0.37-0.58) 0
MUT and CNV 127 0.71 (0.55-0.9) 0.00471
CNV Only 130 1.26 (1-1.58) 0.04958
Female 1058 Reference
Male 772 1.31 (1.15-1.49) 0.00005
Age at Diagnosis 1830 1.03 (1.02-1.03) 0
0.5 1.0 2.0
Hazard Ratio (95% CI)
D
Lung Cancer Survival in Patients with EGFR Mutations (age < 60)
n HR CI p
MUT Only 49 Reference
MUT and CNV 8 1.17 (0.36-3.78) 0.79013
Treated with MAB 45 0.43 (0.16-1.16) 0.0969
Treated with TKI 47 1.03 (0.29-3.71) 0.95937
Female 41 Reference
Male 16 0.83 (0.34-2.05) 0.68705
Age at Diagnosis 57 0.99 (0.92-1.08) 0.89445
0.0 0.5 1.0 2.0 3.0
Hazard Ratio (95% CI)
E
Lung Cancer Survival in Patients with EGFR Mutations (age >= 60)
n HR CI p
MUT Only 71 Reference
MUT and CNV 19 0.81 (0.37-1.74) 0.58731
Treated with MAB 52 0.63 (0.34-1.19) 0.15591
Treated with TKI 78 0.65 (0.17-2.39) 0.51367
Female 66 Reference
Male 24 1.7 (0.84-3.44) 0.1374
Age at Diagnosis 90 1.06 (1.02-1.11) 0.00488
0.5 1.0 2.0 3.0
Hazard Ratio (95% CI)

A

Survival Analysis by CNV and Mutation in Colorectal Cancer

APC

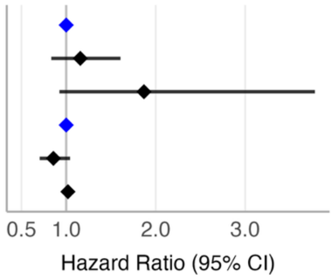

	n	HR	CI	p
MUT Only	861	Reference		
MUT and CNV	93	1.16	(0.83-1.61)	0.38206
CNV Only	18	1.87	(0.92-3.78)	0.08178
Female	448	Reference		
Male	520	0.86	(0.7-1.04)	0.12396
Age at Diagnosis	968	**1.02**	**(1.01-1.03)**	**0.00001**

B

Survival Analysis by CNV and Mutation in Colorectal Cancer

FBXW7

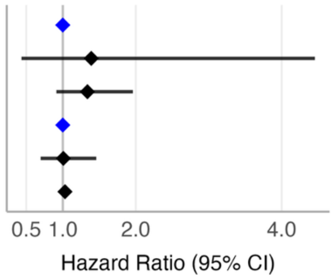

	n	HR	CI	p
MUT Only	184	Reference		
MUT and CNV	6	1.39	(0.43-4.45)	0.57943
CNV Only	101	1.34	(0.91-1.96)	0.13606
Female	132	Reference		
Male	157	1.01	(0.7-1.46)	0.96683
Age at Diagnosis	289	**1.03**	**(1.01-1.05)**	**0.00017**

C

Colorectal - Survival Analysis by CNV and Mutation - All Patients vs PIK3CA

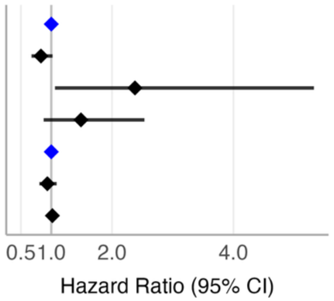

	n	HR	CI	p
No Mut, CNV	1161	Reference		
MUT Only	292	0.83	(0.67-1.02)	0.07727
MUT and CNV	9	**2.38**	**(1.06-5.33)**	**0.03525**
CNV Only	23	1.49	(0.87-2.53)	0.14254
Female	673	Reference		
Male	805	0.94	(0.8-1.09)	0.38402
Age at Diagnosis	1478	**1.02**	**(1.01-1.02)**	**0**

D

Colorectal - Survival Analysis by CNV and Mutation - All Patients vs FBXW7

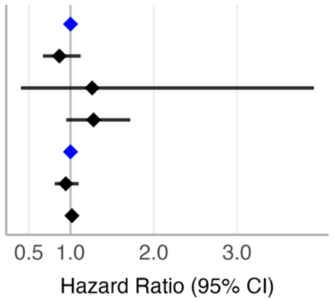

	n	HR	CI	p
No Mut, CNV	1194	Reference		
MUT Only	184	0.87	(0.67-1.12)	0.27589
MUT and CNV	6	1.26	(0.4-3.92)	0.68981
CNV Only	101	1.28	(0.95-1.72)	0.10767
Female	673	Reference		
Male	805	0.94	(0.81-1.1)	0.44619
Age at Diagnosis	1478	**1.02**	**(1.01-1.02)**	**0**

E

Colorectal - Survival Analysis by CNV and Mutation - All Patients vs APC

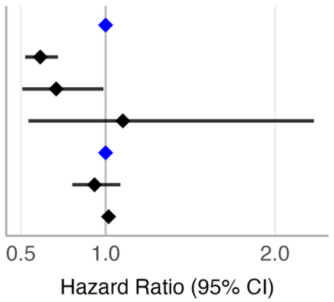

	n	HR	CI	p
No Mut, CNV	513	Reference		
MUT Only	861	**0.61**	**(0.53-0.72)**	**0**
MUT and CNV	93	**0.71**	**(0.51-0.99)**	**0.04191**
CNV Only	18	1.1	(0.54-2.23)	0.78744
Female	673	Reference		
Male	805	0.93	(0.8-1.09)	0.3806
Age at Diagnosis	1478	**1.02**	**(1.01-1.02)**	**0**
